# Emergent Properties of the HNF4α-PPARγ Network May Drive Consequent Phenotypic Plasticity in NAFLD

**DOI:** 10.3390/jcm9030870

**Published:** 2020-03-22

**Authors:** Sarthak Sahoo, Divyoj Singh, Priyanka Chakraborty, Mohit Kumar Jolly

**Affiliations:** 1 Undergraduate Programme, Indian Institute of Science, Bangalore 560012, India; sarthaksahoo@iisc.ac.in (S.S.); divyojsingh@iisc.ac.in (D.S.); 2 Centre for BioSystems Science and Engineering, Indian Institute of Science, Bangalore 560012, India; cpriyanka@iisc.ac.in

**Keywords:** NAFLD, NASH, phenotypic plasticity, mathematical modeling, systems biology, HNF4a, PPARg

## Abstract

Non-alcoholic fatty liver disease (NAFLD) is the most common form of chronic liver disease in adults and children. It is characterized by excessive accumulation of lipids in the hepatocytes of patients without any excess alcohol intake. With a global presence of 24% and limited therapeutic options, the disease burden of NAFLD is increasing. Thus, it becomes imperative to attempt to understand the dynamics of disease progression at a systems-level. Here, we decoded the emergent dynamics of underlying gene regulatory networks that were identified to drive the initiation and the progression of NAFLD. We developed a mathematical model to elucidate the dynamics of the HNF4α-PPARγ gene regulatory network. Our simulations reveal that this network can enable multiple co-existing phenotypes under certain biological conditions: an adipocyte, a hepatocyte, and a “hybrid” adipocyte-like state of the hepatocyte. These phenotypes may also switch among each other, thus enabling phenotypic plasticity and consequently leading to simultaneous deregulation of the levels of molecules that maintain a hepatic identity and/or facilitate a partial or complete acquisition of adipocytic traits. These predicted trends are supported by the analysis of clinical data, further substantiating the putative role of phenotypic plasticity in driving NAFLD. Our results unravel how the emergent dynamics of underlying regulatory networks can promote phenotypic plasticity, thereby propelling the clinically observed changes in gene expression often associated with NAFLD.

## 1. Introduction

The dawn of the 21st century has seen a major global outbreak of obesity and metabolic syndrome. In 2016, the World Health Organization (WHO) estimated that nearly two billion people are either overweight or obese. Primary factors driving this non-communicable epidemic are caloric excess and a sedentary lifestyle, leading to chronic diseases [1]. In the liver, these chronic diseases manifest as non-alcoholic fatty liver disease (NAFLD). It is the most common form of chronic liver disease among adults and children, with a global prevalence of 24% [1,2]. NAFLD is defined as an excessive accumulation of fat in the liver, with at least 5% of hepatocytes exhibiting accumulated triglycerides and without an excess intake of alcohol (daily intake <30 g in men and <20 g in women per day). The disease spectrum varies from benign hepatocellular simple steatosis (SS) of the liver characterized by excessive and abnormal retention of triglycerides and cholesterol esters to a more severe form, called non-alcoholic steatohepatitis (NASH), marked by chronic inflammation of the liver and possibly resulting in cell death and subsequent cirrhosis or fibrosis in the liver. In a small fraction of cases, it can progress to form hepatocellular carcinoma (HCC) [2].

NAFLD is a complex disease that results from an intricate interplay of intertwined genetic and environmental factors [3,4,5]. On one hand, the genome wide association studies (GWAS) have identified variants in the genes PNPLA3, TM6SF2, GCKR, MBOAT7, and HSD17B13 that seem to be associated with susceptibility to and/or progression of NAFLD [6]. A single-nucleotide polymorphism in PNPLA3, causing I to M transition at position 148, remains to be the variant that is most robustly associated with the entire spectrum of NAFLD. The evidence for the heritability of NAFLD is strengthened by data derived from epidemiological, familial aggregation, and twin studies [6]. On the other hand, the prevalence of NAFLD is strongly associated with metabolic syndromes (obesity, type 2 diabetes mellitus, insulin resistance, and dyslipidemia) [7]. Therefore, some recent attempts have even suggested renaming NAFLD as MAFLD (metabolic associated fatty liver disease) [8]. However, the association of NAFLD with obesity need not be universal; particularly, in Asian populations, NAFLD in the absence of obesity—so-called “lean-NAFLD”—has been reported. Lean NAFLD patients may be characterized by different pathogenetic processes as compared to obese NAFLD ones [1,9,10,11,12].

Recently, a molecular-level understanding of NAFLD has been emerging through the identification of signaling pathways implicated in hepatic lipid homeostasis. MAPK, NF-kB, AMPK, and AKT pathways, among others, have been identified to be dysregulated in NAFLD [13]. In NAFLD, the lipids accumulated in the liver are primarily derived from the serum fatty acid (FA) pool (60%), followed by a contribution from de-novo lipogenesis (DNL) (25%; three-fold higher than healthy controls) and the remaining 15% from the dietary sources. Many genes involved in adipogenic programs play a key role in the initiation and the progression of NAFLD [7]. Peroxisome proliferation-activated receptor gamma 2 (PPARγ), a master regulator of adipogenesis [14], is frequently upregulated in NAFLD [15]. Similarly, sterol regulatory binding element protein-1c, SREBP-1c (protein coded by the gene SREBF1), a major driver of hepatic DNL, is upregulated in NAFLD [7]. Consequently, many coregulators and downstream target genes of PPARγ and SREBP-1c are also perturbed in the context of NAFLD [15]. Another intriguing observation about NAFLD patients is their lower levels of hallmark liver tissue maintenance genes such as hepatocyte nuclear factor 4α (HNF4α) and hepatocyte nuclear factor 1α (HNF1α) [16,17,18]. HNF4α is an established master regulator of induction and maintenance of the hepatic cell state [19,20]. HNF4α knockout mice exhibit severe hepatomegaly (enlarged liver) and steatosis [16,19]. Similar to HNF4α-knockout mice, HNF1α knockout also leads to a fatty liver phenotype with increased fatty acid synthesis and steatosis in the liver [21].

The abovementioned studies identify various players associated with NAFLD in a correlative and/or causative manner. However, most studies focus on investigating how the upregulation or the downregulation of one or a pair of these genes affect the final phenotype of the disease. Thus, the emergent dynamics of disease initiation and progression as a consequence of interactions among these genes in a regulatory network is not well understood. Consequently, it still remains elusive how these coordinated gene expression changes emerge as a result of the dynamics of underlying regulatory networks.

Here, we identified a core regulatory network involving HNF4α, HΝF1α, PPARγ, and SREBP-1c and show how interconnections among these key players can drive NAFLD. Our results highlight that this network can give rise to multi-stability, i.e., the co-existence of multiple phenotypes—hepatocyte state (high HNF4α, low PPARγ), adipocyte state (low HNF4α, high PPARγ), and a “hybrid” adipocyte-like state of the hepatocytes (high HNAF4a, high PPARγ). Our results show that, during NAFLD, cells can switch their phenotype from being a hepatocyte to a hybrid adipocyte-like state and *vice versa*, thus controlling phenotypic plasticity in the context of NAFLD. The clinical data from NAFLD patients support the trends as predicted from the model, i.e., deregulated levels of HNF4α, HΝF1α, PPARγ, and SREBP-1c. These results offer important insights into the emergent systems-level dynamics of the regulatory network driving NAFLD.

## 2. Materials and Methods

### 2.1. RAndom CIrcuit PErturbation (RACIPE) Analysis:

#### 2.1.1. RACIPE Simulations

RAndom CIrcuit PErturbation (RACIPE) is a computational method to discern the robust dynamical properties of a particular gene regulatory network topology. It takes in a network topology file as an input and then samples 10000 different sets of parameters. For each parameter set, RACIPE chooses a random set of initial conditions (n = 100) for each node in the network and solves, using Eulers’ method, with the set of coupled ordinary differential equations (ODEs) that represent the interactions among the different nodes in a network. For each given parameter set and initial conditions, RACIPE reports the steady-state values for each of the nodes in the network.

**Figure 1:**
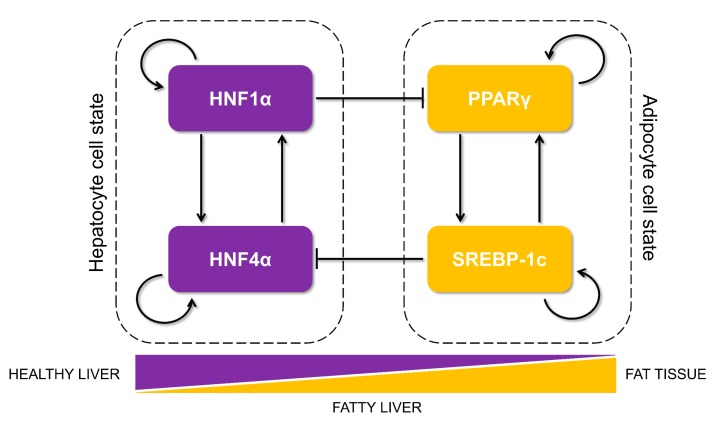
Proposed core regulatory network motif that controls lipid homeostasis in the liver and is dysregulated during the progression of non-alcoholic fatty liver disease (NAFLD). The motif is composed of two tightly regulated, self-activated pairs of transcription factors (each pair shown in a dotted rectangle): HNF4α/HNF1α (maintaining the hepatocyte cell state) and PPARγ/SREBP-1c (driving an adipocytic cell state and implicated in initiation and progression of NAFLD). The arrows show transcriptional activation, and the solid bars represent transcriptional repression. The relative levels of these proteins in a cell can determine its cell state as hepatocytes in healthy liver, adipocytes in the fat tissue, or steatotic hepatocytes in livers of patients suffering from fatty liver.

The parameters are sampled randomly from a specified predefined parameter range for the set of ODEs. We ran RACIPE on our core gene regulatory network shown in Figure 1, using the default parameters given in RACIPE algorithm but limiting the maximum number of states possible to 4. The reason for setting this limit to 4 was that only 7.58 ± 0.08 % of parameter sets enabled > 4 states from the initial analysis using RACIPE.

Note that, since we were considering one variable for each node simulated without treating the mRNA and protein levels separately, we named the genes in all caps to represent this variable throughout the study (HNF4A for HNF4α; HNF1A for HNF1α; PPARG for PPARγ; and SREBF1 for SREBP-1c). The RACIPE results shown for this network remain largely invariant irrespective of the integration method (Euler vs. Runge–Kutta)/number of initial conditions (n = 100, 1000, 1000) chosen for every parameter set (Figure S6B–D).

#### 2.1.2. Z-Score Normalizations of The Steady State Values

RACIPE provides the steady state values in log2 scale. We performed z-score normalizations on steady state values that were obtained from the RACIPE simulations so that the distributions/expression values from various nodes could be compared to one another. To compute the z-score transformed expression value for each steady state value ($E_{i}$) of a given node, we first collated all the untransformed expression values for that node, n. We then computed the mean (${\bar{E}}_{n}$) and the standard deviation ($\sigma_{n}$) for this list of untransformed expression values. Then, each of the steady state values were transformed by the following formula:

(1) $$z_{i}=\frac{E_{i}-{\bar{E}}_{n}}{\sigma_{n}}$$

where $z_{i}$ is the z-normalized expression value. On plotting the distributions, we found that it was largely bimodal, and the corresponding z-score value at the central minima of the distribution could segregate the values into two groups, which we termed as high (H) and low (L).

#### 2.1.3. Density Plots, Bimodality Coefficients, and Clustering Analysis:

For each node, we plotted the distribution of the z-normalized expression values as a Kernel Density Estimate (KDE) curve. This produced a smoothened curve for the distribution as a probability density function for a finite sample size. We also computed Sarle’s bimodality coefficient for finite samples using the following formula:

(2) $$b=\frac{g^{2}+1}{k+\frac{3{\left(n-1\right)}^{2}}{\left(n-2\right)\left(n-3\right)}}$$

where n is the number of items in the sample, g is the sample skewness, and k is the sample excess kurtosis. As the value of b for the uniform distribution was 5/9, values greater than 5/9 indicate a multimodal distribution (in our case, bimodal). We performed unsupervised hierarchical clustering of the heatmaps, which yielded 4 predominant clusters. For clustering analysis for the scatter plots of a pair of genes, we used hierarchical agglomerative clustering by fixing the number of clusters that were possible to 4 (H or L for each of the two nodes, hence a maximum of 4 possible clusters).

### 2.2. Relative Stability Analysis

To perform a relative stability analysis of a given phase, i.e., coexisting combination of phenotypes such as {HL, LH}, {HL, LH, LL}, etc., we collected all the parameters sets that produced that particular phase from the RACIPE analysis. We then explicitly simulated the set of ODEs used in RACIPE in MATLAB for 1000 different initial conditions chosen randomly and sampled from a uniform log2 scale. We plotted a Kernel density estimate for the distribution of the fraction of the cases for which a particular state was achieved for the given phase.

### 2.3. Dynamic Simulations

#### 2.3.1. Bifurcation Diagrams

To plot the bifurcation diagram to show a switch in the levels of HNF4A and PPARG, we used the MATLAB based software MATCONT to simulate the gene regulatory network using a set of ordinary differential equations (ODEs). We used the following set of differential equations, where the rate of change of the expression levels of each node has two terms: a production term (also includes the regulation of that particular node by other nodes) and a degradation term (assumes first order kinetics). Each interaction (regulation term) in the gene regulatory network between a pair of nodes is represented by a shifted Hills function (H_S_), where H_S_ for an interaction of B affecting the production of A is defined as:

(3) $$Hs\left(B,\lambda ,n\right)=H^{-}\left(B\right)+\lambda H^{+}\left(B\right)$$

(4) $$H^{-}\left(B\right)=\frac{B_{0}{}^{n}}{B_{0}{}^{n}+B^{n}}$$

(5) $$H^{+}\left(B\right)=1-H^{-}\left(B\right)$$

where B_o_ is the threshold value for that interaction, n is the cooperativity for that interaction, and λ is the fold change from the basal synthesis rate of A due to B. Therefore, λ > 1 for activators and λ < 1 for inhibitors.

The set of equations that were used to simulate the core gene regulatory network are as follows (green: maximal production rate; cyan: regulation term; yellow: degradation term):(6)
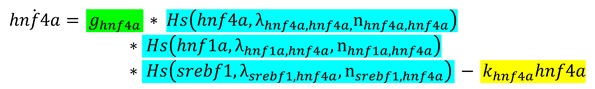

(7)
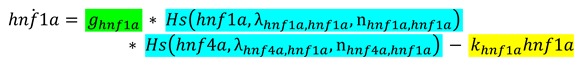

(8)
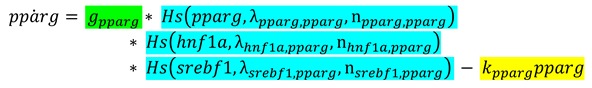

(9)
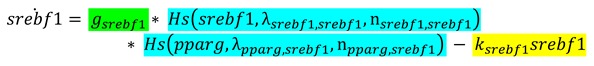


We used the degradation rate of PPARG as the bifurcation parameter. Refer to the Table 1 and Table 2 for the parameter values used for the simulations.

The degradation rates were calculated from the reported half-life values of the corresponding proteins assuming first order kinetics, i.e., degradation rate = ln2 / t_1/2_.

#### 2.3.2. Switching of States

To identify the possibility of switching among states (phenotypes), we added an additional noise term to each of the equations. The noise term added was a random number generated from a Gaussian distribution and multiplied by an amplitude/scaling factor. The amplitude of noise for HNF4A, HNF1A, and SREBF1 were kept at 0.1, mimicking the intrinsic noise present in the system, and 15 for PPARG, mimicking the combined effect of intrinsic noise and extrinsic noise due to a burden of fatty acids to the cells. The abovementioned set of differential equations was updated as follows: (green: maximal production rate; cyan: regulation term; yellow: degradation term; red: noise term):(10)
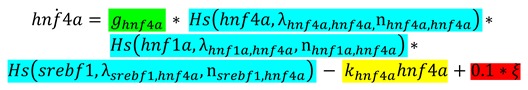

(11)
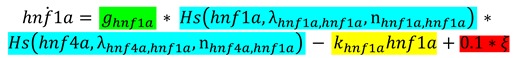

(12)
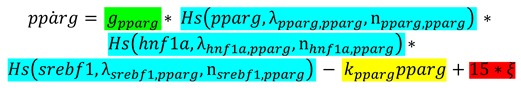

(13)
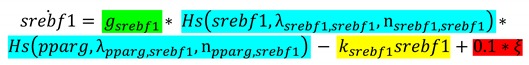

where ξ is a random number picked from a normal distribution with a mean of 0 and a standard deviation of 1. This set of equations (10-13) was solved explicitly in MATLAB using the ode45 solver.

### 2.4. Randomization of Networks

We created an ensemble of all randomized “hypothetical” networks possible using the following rules: for each node, in each instance of randomization of the wild type network (Figure 1), the indegree and the outdegree of the network were kept fixed. The number of activation edges and the number of inhibitory edges in the entire network were also kept fixed at 8 and 2, respectively (the same number as that in the wild type network (Figure 1). Furthermore, the source node and the target node for each of the edges were kept fixed, but the identity of the edge in terms of it being an activation or inhibition link was allowed to change. Hence, 44 (C210−1=10!8!2!−1) such randomized “hypothetical” networks were constructed, excluding the wild type case.

### 2.5. Jensen–Shannon Divergence (JSD) and Plasticity Scores

For each of the randomized and the wild type networks, we calculated the Jensen–Shannon divergence (JSD) score as follows. We first simulated each of the randomized networks along with the wild type network via RACIPE to obtain the steady state solutions that were possible on a set of 10000 randomly chosen parameter sets. We performed z-score normalizations on the obtained steady state solutions and binarized the expression levels of each of the four genes as high (H) or low (L), as mentioned in the methods part of RACIPE analysis. We then constructed a frequency distribution of all the possible states across mono-stable and multi-stable parameter sets (state frequency distribution) and compared each of the distributions to the reference distribution of the wild-type network to get a corresponding JSD score. All possible 16 (=2^4^) states emerging from considering the levels of all four nodes in the network were chosen to calculate the JSD.

In short, for any two discrete frequency distribution P(x) and Q(x),

(14) $$\mathrm{JSD}(P||Q)\;\mathrm{is}\;\mathrm{defined}\;\mathrm{as}\;\frac{1}{2}D(P||M)+\frac{1}{2}\;D(Q||M)$$

(15) $$\mathrm{where}\;M=\frac{1}{2}\left(P+Q\right)$$

and D stands for the Kullback–Liebler divergence and is defined as:

(16) $$D(P||Q)=\underset{x\neq \chi }{\sum }P\left(x\right)\log\left(\frac{P\left(x\right)}{Q\left(x\right)}\right)$$

JSD varies from 0 to 1 where 0 corresponds to an identical distribution, whereas 1 corresponds to no overlap between the distributions (Figure S6A). This implies that the smaller the JSD is, the closer the state frequency distribution of the randomized “hypothetical” network is similar to the “wild type” network.

The quantification score (a proxy for the level of plasticity enabled by a gene regulatory network) is defined as:

(17) $$P\;=\frac{\;N\left(\mathrm{multi}\right)}{N\left(all\right)}=1-\frac{N\left(mono\right)}{N\left(all\right)}$$

where:

N (multi) = number of parameter sets enabling multi-stable solutions;

N (mono) = number of parameter sets enabling mono-stable solutions and;

N (all) = total number of parameter sets considered.

The number of parameters sets that enabled mono-stability or multi-stability were obtained from RACIPE analysis carried on the circuit of interest.

### 2.6. Clinical Data Analysis

We obtained publicly available transcriptomic data for healthy controls and patients suffering from NAFLD/NASH. We downloaded the preprocessed transcriptomic data from each study, and log2 normalized the expression levels if they were not normalized already. The expression values were then used to plot the correlation plots or for comparison of levels of the different genes.

### 2.7. Statistical Tests and Correlation Coefficients

We computed the Spearman correlation coefficients and used the corresponding p-values to gauge the strength of correlation in their expression values between a pair of genes. For statistical comparison values used throughout the study, we used a two-tailed Student’s t-test and computed its significance.

## 3. Results

### 3.1. Identification of a Core HNF4α-PPARγ Network in Hepatocytes

As a first step, we gathered experimentally curated information about interconnections among HNF4α, HΝF1α, PPARγ, and SREBP-1c in the context of lipid homeostasis in the liver and its disruption during the progression of NAFLD. HNF4α and HNF1α were identified as those among the six master regulators for human hepatocytes using chromatoimmuno-precipitation and high-resolution promoter microarrays [29]. Both of them can activate each other as well as positively auto-regulate themselves [18,29]. Their expression levels are lower in fatty liver disease patients as compared to healthy controls [16]. HNF4α plays a crucial role in hepatic development as well as in hepatic lipid homeostasis. Mouse embryos deficient in HNF4α can initiate liver development but not transcriptionally activate the liver-specific genes [30]. Further, adult mice lacking hepatic HNF4α (Hnf4-LivKO) tend to have disrupted lipid homeostasis in vivo and display a fatty liver phenotype [31,32]. Interestingly, the liver of Hnf4-LivKO mice had higher levels of mRNA and protein of PPARγ as compared to the liver of wild-type mice [32]. Recent studies revealed HNF1α as a direct transcriptional repressor of PPARγ in the context of hepatic steatosis [26].

PPARγ and SREBP-1c, on the other hand, are two master regulators of adipocytic cell-fate. PPARγ is necessary and sufficient for adipogenesis in mammals [14]; overexpression of PPARγ has been reported to induce adipogenesis [33]. SREBP-1c, the major transcriptional regulator of lipogenesis, induces the cohort of genes necessary for synthesizing fatty acids [34] and increases DNL in the liver, a hallmark of NAFLD [35]. SREBP-1c is induced during the differentiation of 3T3-L1 preadipocytes [36]. Both PPARγ and SREBP-1c are upregulated in obese patients with NAFLD [37]; they can both self-activate directly or indirectly [38,39,40,41] and augment each other [27,28,42,43]. SREBP-1c can transcriptionally activate PPARγ directly by binding to a putative E-box in the promoter of PPARγ [42] and can increase the transcriptional activity of PPARγ indirectly via the production of ligands for PPARγ [27]. Similarly, SREBF1 is predicted to be a high confidence target of PPARγ [43]. Intriguingly, SREBP-1c has been reported to transcriptionally repress HNF4α in vitro and in vivo in the rodent liver [28]. It can also inhibit the transcriptional activity of HNF4α, suppressing the expression of hepatic gluconeogenic genes [44].

The abovementioned interactions put together results in a core regulatory circuit (Figure 1) that consists of two pairs of closely connected and self-activating transcription factors and a mutual inhibition between these two pairs. One pair (HNF4α and HNF1α) drives the hepatocytic cell state, while the other pair (PPARγ and SREBP-1c) drives the adipocyte cell state. A mutual inhibition between these pairs is reminiscent of a “toggle switch” seen between many “master regulators” of two (or more) divergent cell fates, as identified during multiple instances during embryonic development and disease progression [45,46].

### 3.2. The Emergent Properties of This Core Regulatory Network Enable The Existence of Multiple Phenotypes

Next, we investigated the dynamics emerging from this regulatory network. To characterize the robust dynamical properties of this network, we used a recently developed computational method, RAndom CIrcuit PErturbation (RACIPE) [47]. Given a topology of a regulatory network, RACIPE generates an ensemble of kinetic/biochemical models corresponding to the network topology and later utilizes statistical tools to identify the robust dynamic properties emerging from the specific network topology. For each model in the ensemble, this method samples kinetic parameters from a biologically relevant chosen range of values. Thus, each kinetic model simulated via RACIPE takes a unique combination of parameters with the goal of capturing cell-to-cell heterogeneity in the biochemical reaction rates. An ensemble of these models thus denotes the behavior of a cell population, where each cell contains the given network but has different kinetic parameters from one another.

**Figure 2:**
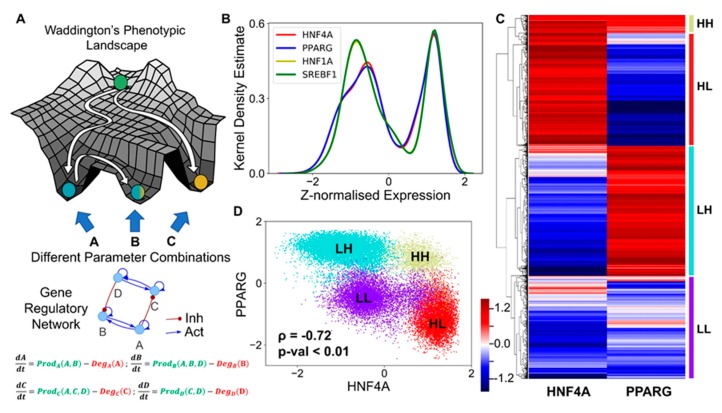
Core regulatory network may enable multiple phenotypes. (**A**) Schematic of Waddington’s phenotypic landscape highlighting the possibility of multiple states as a result of the underlying gene regulatory network, the dynamics of which can be modeled by a set of ordinary differential equations representing the interactions among them as shown below. (**B**) Kernel density plot showing the bimodal distribution of each component of the network, implying that each of the transcription factors (nodes) can exist in either a high (H) or a low (L) state. The x-axis shows the z-normalized log2 expression values of the given component from RACIPE analysis. (**C**) Heatmap showing the relative levels of HNF4A and PPARG and the resulting four clusters. The color bar represents the relative levels of the individual components (z-normalized log2 expression values). (**D**) Scatter plot showing the existence of the four distinct clusters (defined based on hierarchical clustering) as the four states based on relative levels of HNF4A and PPARG (z-normalized log2 expression values). Spearman’s correlation was performed to obtain the correlation coefficient (ρ) and the corresponding p-value (p-val).

Here, each kinetic model is a set of four coupled ordinary differential equations (ODEs), each of which tracks the temporal changes in the levels of the four players (HNF4α, HΝF1α, PPARγ, and SREBP-1c) present in the core regulatory circuit. Each of these four players has a rate of production and a rate of degradation; the production rate is governed by transcriptional regulation from other players (for instance, HNF4α is repressed by SREBP-1c) captured via Hills function [48], while the degradation term assumes first order kinetics and is independent of the levels of all the other genes). The set of differential equations are then solved numerically to attain the steady state values for each of the regulated players. These values are then mapped onto possible biological phenotypes represented as an attractor in the Waddington’s landscape [49] of cell fate determination (Figure 2A). For each given parameter set, depending on the initial condition, each of these molecular players can converge to one of the many possible steady states enabled by that parametric combination. Thus, the circuit considered here can be possibly multi-stable, potentially enabling phenotypic plasticity and/or heterogeneity.

We collated the levels of HNF4α, HΝF1α, PPARγ, and SREBP-1c obtained from all parameter combinations and plotted the distribution of these levels. The distribution of levels of each of these four players was observed to be largely bimodal (Figure 2B, Figure S1A), as measured via Sarle’s bimodality coefficient [50], suggesting that each of these players could exist in either a “high” or a “low” state. Given that HNF4α and PPARγ are the key regulators of the hepatic and the adipocytic state, respectively, we plotted the levels of HNF4α and PPARγ as a clustered heatmap. This analysis revealed four clusters corresponding to four different phenotypes: HNF4α-high and PPARγ-high (HH), HNF4α-low and PPARγ-low (LL), HNF4α-low and PPARγ-high (LH), HNF4α-high and PPARγ-low (HL) (Figure 2C). Interestingly, the HL and the LH clusters were found to be the most abundant (Figure S2A), suggesting that this regulatory network can exist in two predominant states—HL (HNF4α-high, PPARγ-low) and LH (HNF4α-low, PPARγ-high). These states correspond to the hepatocyte and the adipocyte phenotype, respectively. Biologically speaking, this result is consistent with experimental observations that the exogenous overexpression of either HNF4α or PPARγ can drive the cell fate to a hepatocyte (HL) or an adipocyte (LH), respectively [33,51], where the level of the other master regulator is relatively quite low (Figure S2B), i.e., either HNF4α/PPARγ >>1 or HNF4α/PPARγ << 1. Besides these two states, the network can exist in either the HH (HNF4α-high, PPARγ-high) or the LL (HNF4α-low, PPARγ-low) state. The HH state can possibly be mapped onto the “hybrid” adipocyte-like phenotype of the hepatocytes, where the levels of both HNF4α and PPARγ are high, similar to observations made in other scenarios where co-expression of mutually opposing master regulators have been reported [52,53]. The LL state can be thought of as an uncommitted “stem-like” phenotype where the levels of both master regulators are low, and hence it is not committed to either of the cell lineages. These observations remain qualitatively unchanged, even when all the four regulatory players are considered for clustering (Figure S1G).

Consistent with the predominance of HL and LH phenotypes, the scatter plot of all steady state solutions revealed a significant negative correlation between HNF4α and PPARγ (Figure 2D), indicating that, while cell-to-cell heterogeneity may enable varying levels of HNF4α and PPARγ in a cell population, these levels remain anti-correlated at a population level. Consistently, HNF4α levels correlated positively with those of HNF1α but negatively with SREBP-1c, and PPARγ levels correlated positively with those of SREBP-1c but negatively with HNF1α (Figure S1B–F). Put together, these results imply that the core regulatory circuit among HNF4α, HΝF1α, PPARγ, and SREBP-1c can give rise to multiple cell fates in a biologically relevant parameter regime, with the more frequent states being a hepatocyte (HL—HNF4α-high, PPARγ-low) and an adipocyte (LH—HNF4α-low, PPARγ-high).

### 3.3. Multiple Stable States (Phenotypes) Can Co-Exist, Giving Rise To Phenotypic Plasticity

**Figure 3:**
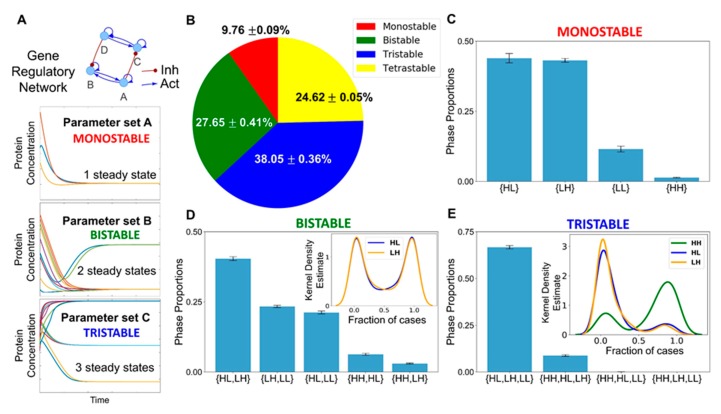
Co-existence and relative stability of multiple phenotypes giving rise to multi-stable phases. (**A**) Schematic showing how a gene regulatory network for different parameter sets end up in a mono- or a multi- (bi-, tri-) stable regime. Depending on different initial conditions, the system (levels of the different regulators) can converge to one or more steady state level(s), enabling mono- or multi-stability. (**B**) A pie chart showing the fraction of parameter sets giving rise to mono-, bi-, tri-, and tetra-stability (mean ± standard deviation over three independent replicates through RACIPE analysis). (**C**) Bar plot showing the proportions of the various phases of the monostable solutions, namely, {HL}, {LH}, {LL}, and {HH}. (**D**) Bar plot showing the proportions of the various phases possible for bi-stable solutions. The kernel density estimate plot in the inset shows the relative stability of the HL and the LH states for the phase {HL, LH}. (**E**) Bar plot showing the proportions of phases possible for the tri-stable solutions. The kernel density estimate plot in the inset shows the relative stability of HL, LH, and HH states for the phase {HL, LH, HH}. Error bars represent the standard deviation of the mean values of the phase proportions over three independent RACIPE replicates. Panels B–E are based on three replicates of RACIPE simulations on the network shown in Figure 1.

Next, we investigated the possibility of whether two or more steady states (phenotypes) can co-exist, thus possibly enabling phenotypic plasticity. In the ensemble of parameters sets simulated via RACIPE, we found instances where this network led to only one phenotype (mono-stable) as well as instances where it led to two (bi-stability), three (tri-stability), or four (tetra-stability) phenotypes (Figure 3A). Multi-stability (i.e., the co-existence of more than one steady state) can enable cells to switch their phenotypes spontaneously (i.e., without the necessity of any strong external perturbation) depending on the levels of intrinsic or extrinsic biological noise [54,55]. Interestingly, the percentage of parameter sets that gave rise to mono-stability was the least (Figure 3B), suggesting that this core regulatory network is more likely to be multi-stable and thus enable phenotypic plasticity in the context of NAFLD. Among the monostable solutions, HL and LH—corresponding to hepatocyte and adipocyte phenotypes—were the most predominant ones (Figure 3C), reminiscent of our previous observations. Further inspection of the bi- and the tri-stable solutions also revealed a similar trend. Among six (number of ways to choose two out of four solutions) possible bi-stable phases (i.e., combinations of co-existing states), the most frequent phase was {HL, LH}, i.e., co-existence of HL and LH states (Figure 3D). The next two most frequent phases included either HL or LH as one of the states. Similarly, among the four possible tri-stable phases, the two more frequent ones contained both HL and LH, together with either LL or HH, such as {HL, LH, LL} or {HL, LH, HH} (Figure 3E).

Within each phase, there may be varied stability of different co-existing phenotypes. Thus, we quantified the relative stability of the co-existing states in the different multi-stable phases. We calculated the fraction of randomly chosen initial conditions that converged to a particular state for all the parameter sets that enabled multi-stability (bi-, tri-, or tetra-stability). Such calculation can provide insights into how likely the system will attain a particular state for an ensemble of randomly chosen initial conditions and hence can serve as a proxy measurement for the relative stability of that state in that phase. For instance, for a given parameter set corresponding to the phase {HL, LH}, depending on the sampling of initial conditions (say n = 100), x of them can converge to HL state, while (100-x) converge to LH. We calculated the values of fractions of initial conditions leading to HL or LH and plotted the distribution of these values (Figure S3A–C). We found that both the HL and the LH were equally likely to be attained if the system was left to start from a large set of randomly chosen initial levels of the different nodes present in the gene regulatory network when analyzed across parameter sets corresponding to {HL, LH} (Figure 3D inset). Such symmetry in the relative stability of states may emerge due to the symmetry in the network itself (see the two sides of the network in Figure 1). For the phases {HH, LH} and {HH, HL}, the state HH seemed relatively more stable than either LH or HL; conversely, for the phases {HL, LL} and {LH, LL}, the state LL was found to be relatively less stable than HL or LL (Figure S3D–G).

The relative stability trends for the two tristable phases, {HL, LH, LL} and {HL, HH, LH}, and the tetrastable phase, {HL, LH, LL, HH}, reinforced our previous observations. LL was much less stable relative to HL or LH in the phase {HL, LH, LL} (Figure S3H), thus implying that this phase can be effectively considered to be equivalent to a bistable phase {HL, LH}. A possible interpretation would be that LL corresponds to a “stem-like” cell state that is inherently less stable and can quickly differentiate to an adipocyte or a hepatocyte. Contrary to this, for the phase {HL, LH, HH}, the relative stability of the HH state was much larger than that of HL or LH states (Figure 3E inset). The HH state can be thought of as a “hybrid” adipocyte/hepatocyte state, similar to those observed in other tristable cell fate decision networks [53,56,57]. This result, together with similar observations for the tetrastable phase (Figure S3I), emphasizes that a hybrid adipocyte-like state of hepatocytes (i.e., HH) may be prevalent in driving NAFLD.

Together, RACIPE analysis for this core regulatory network reveals a robust feature of this network topology—it not only allows cells to exist in more than one phenotype (HL, LH, HH) but also can facilitate the stability of the HH state, which may be quite relevant during the progression of NAFLD, i.e., a scenario where hepatocytes have not completely lost their identity but do simultaneously express various adipocytic markers and/or traits. The co-existence of HH with other states (HL, LH) raises the possibility that, during the initiation and/or the progression of NAFLD, hepatocytes may reversibly and dynamically switch to this adipocyte-like or hybrid adipocyte/hepatocyte phenotype, emphasizing the role of phenotypic plasticity in NAFLD.

**Figure 4:**
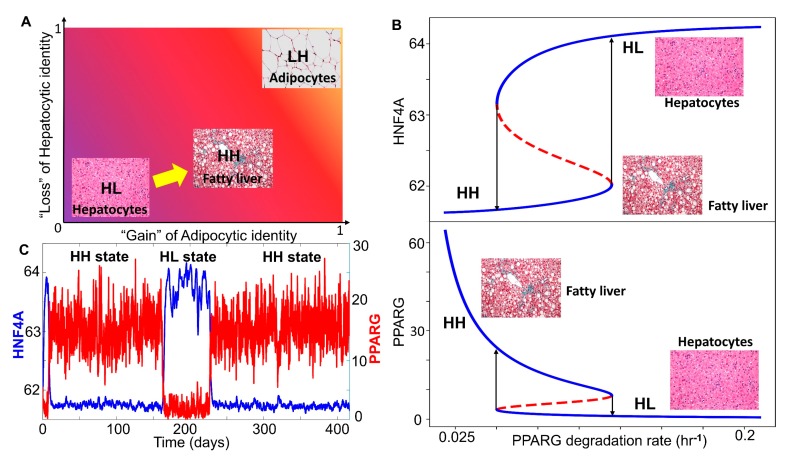
NAFLD as a bistable system involving a phenotypic switch from a hepatocyte to a “hybrid” adipocyte-like phenotype of the hepatocytes. (**A**) Schematic showing the phenotypic transition that may happen in the context of NAFLD where the disease can be highly diverse in terms of the extent of loss of hepatocytic and/or gain of adipocytic characteristics. (**B**) Bifurcation plots showing the phenotypic transitions, i.e., switch in the levels of HNF4A (top panel) and PPARG (bottom panel) when the degradation rate of PPARG is used as a bifurcation parameter. Solid blue lines denote the stable states, dotted red lines denote the unstable states of the system. Arrows indicate transitions between the two states. (**C**) Stochastic simulations of the core gene regulatory network showing switching in HNF4A and PPARG levels (degradation rate of PPARG = 0.08 hr^−1^). (Image credit for “hepatocyte”, “fatty liver”, “adipocytes” in A and B: Wikimedia Commons).

After getting insights into robust dynamical properties of the core regulatory network across a range of parametric combinations, we studied what might possibly be driving NAFLD in a more realistic situation. NAFLD can be construed as a spectrum of diseases where hepatocytes can lose their hepatocytic identities to varying degrees and/or gain adipocytic identities to different extents enabled by phenotypic plasticity of the underlying biological network (Figure 4A). Thus, the histopathological state of the liver seen in NAFLD is more likely to map onto the hybrid HH state, instead of an adipocytic (LH) state. Thus, we hypothesized that NAFLD may progress via a switch from the HL (hepatocyte) state to the HH state (hybrid or adipocyte-like state of hepatocyte).

We estimated parameters (see Materials and Methods) from relevant experimental literature to estimate the typical values of half-lives of proteins and their concentrations in mammalian cells. These parameters were used to calibrate our model and to identify whether a phenotypic switch is implicated during NAFLD. During NAFLD, the upregulation of Hsp90 levels can suppress the degradation of PPARγ, thus increasing PPARγ signaling [58]. We examined the effect of decreasing the degradation rates of PPARγ, through a bifurcation diagram drawn using MATCONT [59]. Thus, as the degradation rate of PPARγ decreased, the steady state levels of PPARγ, which are generally low in the hepatocytes, increased constantly, which switched beyond a certain threshold in PPARγ (Figure 4B bottom panel). As the degradation rate of PPARγ was varied, HNF4α levels dropped modestly (Figure 4B top panel), indicating that the hepatocytic identity was not lost completely. Thus, at a higher degradation rate of PPARγ, the cell was in a hepatocytic state (solid blue line corresponding to “HL”), while lower degradation rates of PPARγ enabled a switch to hybrid state (solid blue line corresponding to “HH”) (Figure 4B). This process can be viewed as “trans-differentiation” of the cell, i.e., conversion of hepatocytes to hybrid adipocyte-like cell state by simultaneous repression of the original tissue (liver) homeostatic mechanisms and activation of a different tissue-specific (adipose tissue) program. This result is consistent with observations of multiple adipocytic markers reported in histological samples of NAFLD patients [60]. In the liver, such trans-differentiation has also been observed for quiescent hepatic stellate cells that can attain adipogenic or myogenic characteristics depending on the relative abundance of adipogenic or myogenic genes, respectively [61].

We further examined whether this core regulatory network can enable phenotypic switching under the influence of biological noise [62]. To mimic various sources of biological noise, we simulated the system stochastically (see Materials and Methods for details) and observed fluctuations in the levels of both PPARγ and HNF4α. Sometimes, these fluctuations were large enough to trigger a transition from the HL (hepatocyte; HNF4α high-PPARγ low) state to the HH state (hybrid; HNF4α high-PPARγ high) and vice versa (Figure 4C). The stability of the HH state can be assessed by the observation that the system may tend to spend a relatively longer time in the HH state rather than the HL state, at least for these specific parameters. It was encouraging to note that the timescale of switching is in the order of three months, a timescale which excellently corroborates clinical observations that NAFLD patients who adopt aggressive lifestyle changes may reverse NAFLD to a significant degree in one month [63]. Overall, we found that, in the biologically relevant range of parameters under which NAFLD seems to be operating, hepatocytes may undergo a phenotypic switch to a hybrid adipocyte-like state of hepatocyte by partially activating the adipogenic program.

### 3.4. The Topology of the Core Regulatory Network is Designed to Enhance Phenotypic Plasticity

**Figure 5:**
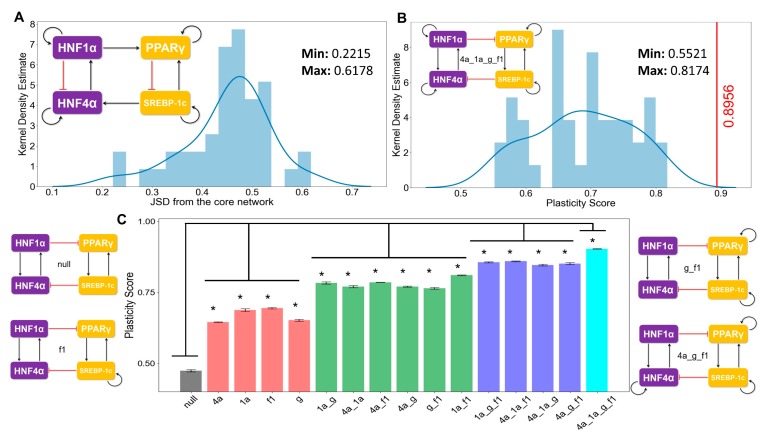
Proposed core regulatory module for NAFLD enables maximum plasticity. (**A**) Kernel density estimate plot with the histogram overlaying showing the distribution of the Jensen–Shannon distance (JSD) between the frequency of state distribution of the “hypothetical” randomized networks from the “wild type” core regulatory network. The maximum and the minimum values of the JSD observed are mentioned in the top right side in the figure. An example of a randomized network is shown at the top left corner. (**B**) Kernel density estimate plot with the overlaying histogram showing the distribution of the plasticity scores of the various random networks. The core regulatory circuit (codenamed 4a_1a_g_f1) is shown in the inset. The red vertical line indicates the plasticity score (=0.89) of the “wild type” core regulatory circuit, which is greater than all other “hypothetical” randomized networks. The maximum and minimum values of the JSD observed are mentioned in the top right side in the figure. Kernel density plots from two other independent RACIPE replicates are included in Figure S4A–D. (**C**) Bar plots showing the plasticity scores of the modified core regulatory networks with a varying number of direct self-activation loops. A few representative examples of the modified (i.e., “mutant”) networks and the corresponding codenames are drawn alongside (null indicates no self-activation loops; 4a, 1a, g, f1 denotes the network topology having self-activations on HNF4A, HNF1A, PPARG, and SREBF1, respectively). * indicates a significant difference of the plasticity scores (p-val < 0.05) when compared via the Student’s t-test with the plasticity of the null network. Error bars denote the standard deviation in plasticity scores calculated for three independent RACIPE replicates.

Next, we investigated how unique the observed traits such as multi-stability were to the topology of the core network underlying NAFLD. In other words, we asked what are the salient features of the topology of core regulatory network formed by reported interconnections among HNF4α, HΝF1α, PPARγ, and SREBP-1c (Figure 1). To discern the effect of network topology, we created an ensemble of randomized networks, where we swapped/shuffled many links in the network with one another while maintaining the number of links that occurred and emanated from all individual nodes in the network. Such randomization enabled us to dissect the contribution of network topology to the above-mentioned network features. For the core regulatory network shown in Figure 1, 44 such “hypothetical” cases are possible (see Materials and Methods). One such example of a “hypothetical” network is shown in Figure 5A inset, where two activatory and two inhibitory links were shuffled with respect to the “wild type” core network topology (Figure 1). In the “wild type” network, HNF1α inhibits PPARγ, and SREBP-1c inhibits HNF4α, while in this “hypothetical network”, HNF1α activates PPARγ, and SREBP-1c activates HNF4α. Moreover, in the “wild type” network, HNF1α activates HNF4α, and PPARγ activates SREBP-1c, but in this “hypothetical” network, HNF1α inhibits HNF4α, and PPARγ inhibits SREBP-1c.

First, we calculated the state frequency of each of the 44 “hypothetical” (i.e., randomized) networks. We then computed the Jensen–Shannon distance (JSD) of the state frequency distribution of each of these “hypothetical” randomized networks from the observed state frequency of the “wild type” (i.e., core) regulatory network. JSD is a measure of distance between two distributions and varies between 0 and 1 [64]. JSD = 0 implies that the two distributions are identical, while JSD = 1 implies that the distributions are completely non-overlapping (Figure S6A). Upon plotting the distribution of the JSD of the 44 “hypothetical” networks, we found that none of the distributions were close to the observed distribution of the “wild type” regulatory network (Figure 5A, Figure S4A,C) (minimum value of JSD = 0.22). This result signifies that the phenotypic distribution attained from the core regulatory network is unique to that network topology.

Next, we quantified the plasticity of the “wild type” network and the 44 “hypothetical” networks. The plasticity of a network is defined to be the fraction of parameter sets that enable multi-stable solutions out of the total number of parameter sets considered (n = 10000 here). We found the “wild type” network to possess the maximum plasticity as compared to each of the 44 “hypothetical” networks (Figure 5B, Figure S4B,D), highlighting that the specific network topology among these master regulators may be optimized to enable maximum phenotypic plasticity.

Self-activation loops are known to contribute largely to the plasticity in many biological systems [65]; thus, we quantified the contribution of different self-activation loops on plasticity. The core network contained four self-activation loops—each for HNF4α, HΝF1α, PPARγ, and SREBP-1c. We created “mutant” networks where one or more self-activation loops were deleted. These networks formed an ensemble of models that covered all possible combinations of self-activation loops; only one, two, or three out of the four nodes had such self-activation loops. We observed that the more self-activation loops there were, the higher the plasticity score was (Figure 5C). Put together, these results underscore that the topology of the core regulatory network plays crucial roles in enabling phenotypic plasticity during NAFLD progression.

### 3.5. Clinical Data Support the Model Predictions

**Figure 6:**
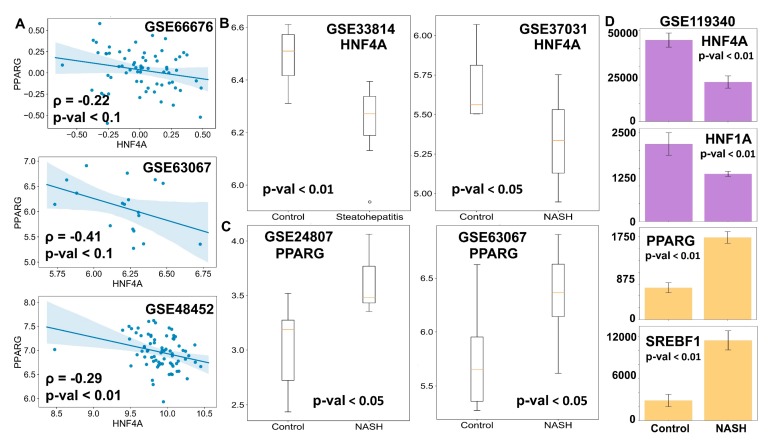
Clinical evidence supports the model predictions. (**A**) Scatter plots between the expression levels of HNF4A and PPARG in clinical samples. Spearman correlation coefficient is given by ρ, and p-val denotes the corresponding *p*-value. (**B**,**C**) Comparison of mRNA levels (log2 normalized) of HNF4A (**B**) and PPARG (**C**) in the liver of NASH patients and healthy controls. (**D**) Comparison of levels of HNF4A, HNF1A, PPARG, and SREBF1 in mouse liver for control case vs. mouse model of NASH. Expression values are listed as TPM (transcripts per million) for given RNA-seq data. *p*-value (p-val) given for Student’s t-test in B–D.

Finally, we tested whether our model predictions about phenotypic plasticity in NAFLD were consistent with the available clinical data. One key prediction of the model was that the expression levels of HNF4α and PPARγ should be negatively correlated (Figure 2D). We observed such trends in multiple clinical datasets (GSE66676, GSE33814, GSE37031) corresponding to NASH and/or NAFLD patients that showed PPARγ and HNF4α to be negatively correlated (Figure 6A). These results indicate that the core programs of hepatocytic and adipocytic identity are inversely correlated. As expected, the levels of HNF4α and HNF1α were found to be positively correlated (Figure S5A), while those of the HNF1α and PPARγ were negatively correlated (Figure S5B).

Next, we examined whether the HNF4α levels were significantly downregulated and/or the PPARγ levels were upregulated in the case of NASH, a more severe stage of fatty liver disease. Indeed, HNF4α was found to be higher in the case of the normal liver than in the case of NASH livers (Figure 6B). Similarly, PPARγ levels were found to be amplified in livers of NASH patients in comparison to healthy livers (Figure 6C). These observations point out that NAFLD progresses via the simultaneous suppression of the hepatic program controlled by HNF4α and the activation of the adipogenic program controlled primarily by PPARγ, although to varying degrees. Consistently, in mouse models of NASH, the liver showed downregulation of HNF4α and HNF1α and up-regulation of PPARγ and SREBP-1c, as compared to controls (Figure 6D). Intriguingly, this pattern was observed for hepatocytes but not in liver endothelial cells, suggesting that this regulatory circuit may be specifically operative in hepatocytes, at least in mouse models (Figure S5C). Put together, these clinical observations strongly support that the proposed core gene regulatory network may underpin phenotypic plasticity in the context of NAFLD initiation and progression.

## 4. Discussion

With the rise of obesity and sedentary lifestyle-induced metabolic disorders, diseases such as NAFLD are on the rise with a very limited number of treatment options available, which are not proven to be very effective [66]. The world has seen a drastic increase in the prevalence of NAFLD in the 21st century, and the number of cases continue to rise. The global incidence of NAFLD and its specific histological phenotype, NASH, has risen dramatically (15% in 2005 to 25% in 2010, and 33% in 2005 to 59.1% in 2010, respectively) [1]. Thus, it becomes imperative to study the initiation and the progression of NAFLD through its various phenotypic stages to devise efficient and robust intervention measures to control the spread of this epidemic. Multiple studies have shown the importance of various key regulators and genetic alterations for the development and the progression of NAFLD [67,68]. However, there are only a few attempts to analyze NAFLD from a systems biology perspective, i.e., decoding how these different regulators interact in the context of NAFLD [17,69,70,71,72]. Thus, mechanism-based modeling studies are required to elucidate these mechanisms from a dynamical systems perspective.

Here, we identified and computationally modeled a core gene regulatory network that appears to be crucial to explain various features of NAFLD. Specifically, we modeled the interactions among two hallmark liver homeostatic transcription factors, HNF4α and HΝF1α, and two master regulators of adipocytic cell fate and lipid homeostasis, PPARγ and SREBP-1c, respectively. Our results show that the dynamical interactions between these transcription factors can drive a hepatocytic or an adipocytic cell fate program in a cell. This core regulatory network is also capable of existing in a “hybrid” state, an adipocyte-like phenotype of the hepatocytes, which might correspond to observations during the progression of NAFLD. This “hybrid” state can be mapped on the presence of large lipid droplets in the hepatocytes with an increased expression of adipocytic markers and enhanced release of various pro-inflammatory cytokines such as IL-6, IL-18, and TNFα, both of which are hallmark features of adipocytes [60]. Our mathematical model also predicts that the hepatocytes can spontaneously switch to form the “hybrid” state under the influence of biological noise. Further, we showed that this core gene regulatory network exhibits higher levels of plasticity both due to its topology and due to the abundance of self-activation loops. Self-activation is frequently seen in biological networks, and its combination with mutually inhibitory circuits, as observed in the context of NAFLD, has been reported to amplify the likelihood of multi-stability and consequent phenotypic plasticity [46,65]. Therefore, targeting the interactions that drive phenotypic plasticity and thus reducing the frequency of switching between hepatocytic and the “hybrid” states can be thought of as a potential therapeutic strategy for NAFLD.

**Figure 7:**
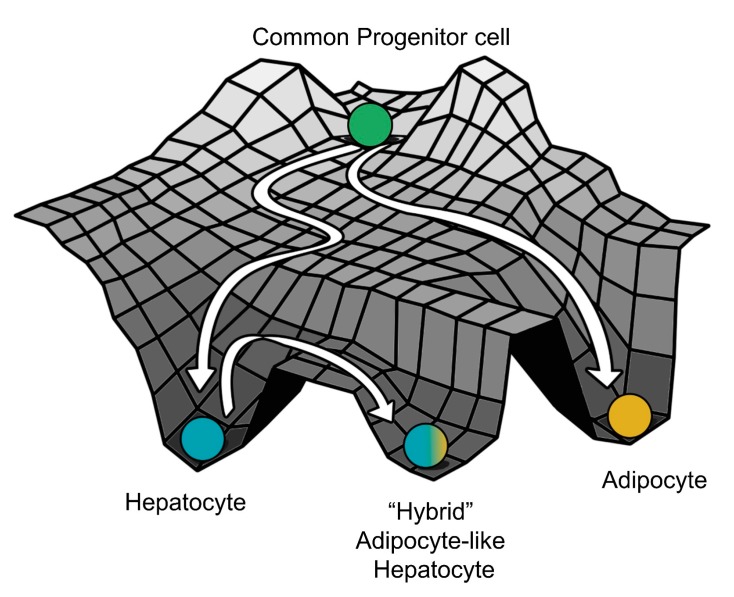
A schematic showing hepatocytes in a fatty liver as a distinct cell state due to “trans-differentiation” from the normal hepatocytes to a much more adipocyte-like “hybrid” cell state on the Waddington’s landscape (The Strategy of the Genes, Waddington C, 1957).

The predictions of our model are also supported by clinical data of patients suffering from NAFLD. Overall, we can construe that the phenotype seen in NAFLD is due to a “trans-differentiation” process, where the cells can switch from a stable attractor state (in this case, the hepatocytes) to another one (in this case, an adipocyte-like “hybrid” state of hepatocytes) (Figure 7). While further work needs to be carried out to elucidate the existence and the extent of phenotypic switching in NAFLD patients, systems biology approaches similar to those presented here can have potential translational implications in terms of sub-phenotyping of patients and guiding subsequent drug repurposing and development [73,74]. For instance, the gut microbiota of lean NAFLD patients is strikingly different from that of obese NAFLD patients; such diverse microenvironmental traits may alter the interactome landscape in NAFLD pathogenesis [75].

Multistability is a hallmark of many cell-fate decision networks [76,77]. Multistable systems often display hysteresis, in other words, an asymmetry in the trajectory of “forward” vs. “backward” responses [57,78,79]. To establish the relevance of multistability in the context of NAFLD, a proposed in vitro experiment would be to catalog the changes in levels of various adipocytic and/or hepatocytic markers at a single-cell scale and in a dose- and/or a time-dependent manner, when hepatocytes are exposed to fatty acids and triglycerides driving NAFLD [80]. Another feature of multistable systems can be “spontaneous switching” among phenotypes under the influence of biological noise [81]. To test this feature, one can isolate a population of steatotic hepatocytes and observe if it can give rise to non-steatotic hepatocytes (implying reversibility) when cultured separately in vitro. The extent of reversibility can depend on various factors such as remodeling of the cellular microenvironment and/or epigenetics [55]. Thus, it would also be interesting to quantify the reversibility of a fatty liver phenotype as the disease progresses from NAFLD to a more inflammatory state, NASH [82]. Specifically, it will be intriguing to examine how various players that are upregulated due to initial phenotypic transition (PPARγ and SREBP-1c) are stabilized due to the activation of the immune response, which is known to be a hallmark of NASH [82,83]. Another important trait that can impact the dynamics of NAFLD is the fluctuations of molecules during the fasting-feeding cycle [84]; for instance, SREBP-1c levels can increase drastically upon fasted mice being refed [85]. It should be noted that, although our model offers a possible explanation for NAFLD progression, such phenotypic switching among adipocytes and hepatocytes has not yet been reported in adult homeostatic conditions given their different developmental lineages and with adipocytes generally believed to be arising from the mesoderm [86], while the hepatocytes arising from the endoderm [87], although functional hepatocytes, have been reported to be generated from human adipose-derived stem cells [88].

There are various limitations of the computational model considered here. First, the network proposed here is by no means exhaustive; various other mechanisms may alter the dynamics of the regulatory network and the consequent relative stability and phenotypic switching. Second, the effect of various genetic variants has not been incorporated into the model at this first step. Third, our current model considers largely transcriptional factors, while both microRNA regulation [89] and post-translational modifications [90] of proteins have been shown to be important in the context of NAFLD. Despite these limitations, this analysis strongly suggests the role of phenotypic plasticity in the development and the progression of NAFLD. It also offers a mechanistic basis for the possible existence of an adipocyte-like phenotype of hepatocytes during NAFLD, where cells may maintain a delicate balance of cellular identity.

## Figures and Tables

**Table 1:** Production rates and degradation rates of different species.

Species	Production Rate (10^6^ molecules hr^−1^)	Degradation Rate (hr^−1^)	References
**HNF4A**	0.4540	0.0491	[22]
**HNF1A**	0.0570	0.0494	[23]
**PPARG**	0.0628	0.08	[24]
**SREBF1**	1.1058	0.1598	[25]

**Table 2:** Activation or inhibition parameters for the interaction.

Description	Fold Change	Value	# of binding sites	Value	Threshold	Value	Reference (est: Estimated)
**Self-Activation of HNF4A**	$\lambda_{hnf4a,hnf4a}$	4	$n_{hnf4a,hnf4a}$	4	$hnf4a_{hnf4a}^{0}$	3	est
**Self-Activation of HNF1A**	$\lambda_{hnf1a,hnf1a}$	2	$n_{hnf1a,hnf1a}$	4	$hnf1a_{hnf1a}^{0}$	0.8	est
**Self-Activation of PPARG**	$\lambda_{pparg,pparg}$	9.514	$n_{pparg,pparg}$	5	$pparg_{pparg}^{0}$	6.320	est
**Self-Activation of SREBF1**	$\lambda_{srebf1,srebf1}$	4	$n_{srebf1,srebf1}$	2	$srebf1_{srebf1}^{0}$	5.25	est
**Activation of HNF4A by HNF1A**	$\lambda_{hnf1a,hnf4a}$	4	$n_{hnf1a,hnf4a}$	3	$hnf1a_{hnf4a}^{0}$	0.8	est
**Activation of HNF1A by HNF4A**	$\lambda_{hnf4a,hnf1a}$	5.328	$n_{hnf4a,hnf1a}$	4	$hnf4a_{hnf1a}^{0}$	5.108	est, [26]
**Activation of PPARG by SREBF1**	$\lambda_{srebf1,pparg}$	3	$n_{srebf1,pparg}$	2	$srebf1_{pparg}^{0}$	5.25	est, [27]
**Activation of SREBF1 by PPARG**	$\lambda_{pparg,srebf1}$	3.729	$n_{pparg,srebf1}$	2	$pparg_{srebf1}^{0}$	9.283	est
**Inhibition of HNF4A by SREBF1**	$\lambda_{srebf1,hnf4a}$	0.415	$n_{srebf1,hnf4a}$	2	$srebf1_{hnf4a}^{0}$	5	[28]
**Inhibition of PPARG by HNF1A**	$\lambda_{pparg,hnf1a}$	0.68	$n_{pparg,hnf1a}$	4	$hnf1a_{pparg}^{0}$	0.674	[26]

The values of λ and n, whenever available, were taken from the above-mentioned references. All other parameters of the model were estimated.

## Supplementary Materials

The following are available online at https://www.mdpi.com/2077-0383/9/3/870/s1, Figure S1: Dynamics of NAFLD core regulatory network, Figure S2: Frequency and characterization of HL and LH states, Figure S3: Relative stability for multistable phases, Figure S4: Kernel density estimate plots, Figure S5: Clinical data estimates, Figure S6: Robustness of methods used.
