# Supplementary material for: Emergent Properties of the HNF4α-PPARγ Network May Drive Consequent Phenotypic Plasticity in NAFLD
Source: J Clin Med. 2020 Mar 22;9(3):870. doi: 10.3390/jcm9030870 (PMC7141525; doi:10.3390/jcm9030870)
Supplement: Supplementary file 1 [file jcm-09-00870-s001.pdf]

SUPPLEMENTARY FIGURES

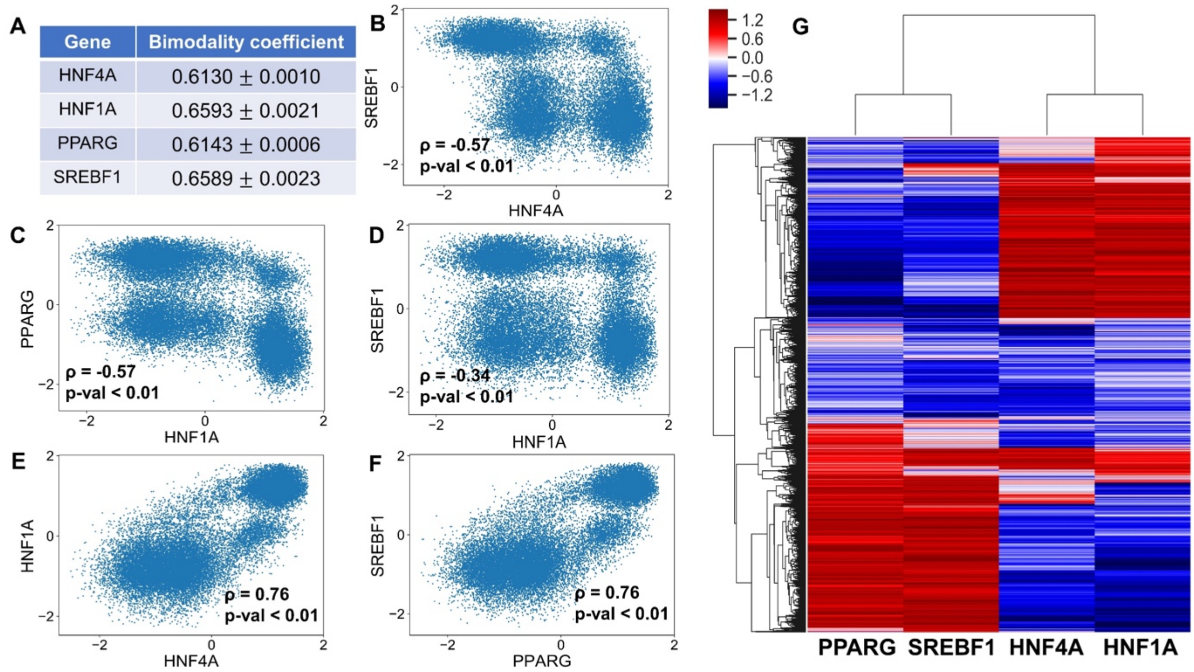

Gene	Bimodality coefficient
HNF4A	0.6130 ± 0.0010
HNF1A	0.6593 ± 0.0021
PPARG	0.6143 ± 0.0006
SREBF1	0.6589 ± 0.0023

FIGURE S1: *Dynamics of NAFLD core regulatory network.* ***(A)*** *Table listing the bimodality coefficients of the four nodes in the core regulatory network over three independent replicates. Mean and standard deviations reflect the statistics over three independent RACIPE replicates.* ***(B-F)*** *Scatter plot showing the relationship between HNF4A-SREBF1, HNF1A-PPARG, HNF4A-HNF1A and PPARG-SREBF1 (z-normalized log2 expression values). Spearman's correlation was performed to obtain the correlation coefficient (ρ) and the significance values (p-val).* ***(G)*** *Heatmap showing the relative levels of all 4 nodes of the core gene regulatory network. The color bar represents the relative levels of the individual components (z-normalized log2 expression values).*

A

State	State frequency
HH	6.53 ± 0.20 %
HL	32.21 ± 0.10 %
LH	35.16 ± 0.11 %
LL	26.09 ± 0.22 %

B

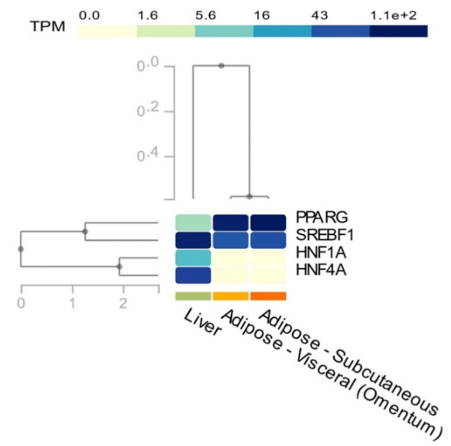

FIGURE S2: *Frequency and characterization of HL and LH states.* ***(A)*** *Table listing the mean percentage of cases that enable the mentioned state along with their standard deviations (mean ± std. dev) across all the possible phases over three independent RACIPE replicates (n=3; each iteration includes 10000 parameter sets).* ***(B)*** *GTEx (Genotype-Tissue Expression) data showing the mRNA expression levels in TPM (Transcripts per million) of the 4 genes of the core regulatory network in Liver and Adipose tissues (Visceral and Subcutaneous) - Data available from GTEx portal on Feb 17, 2020.*

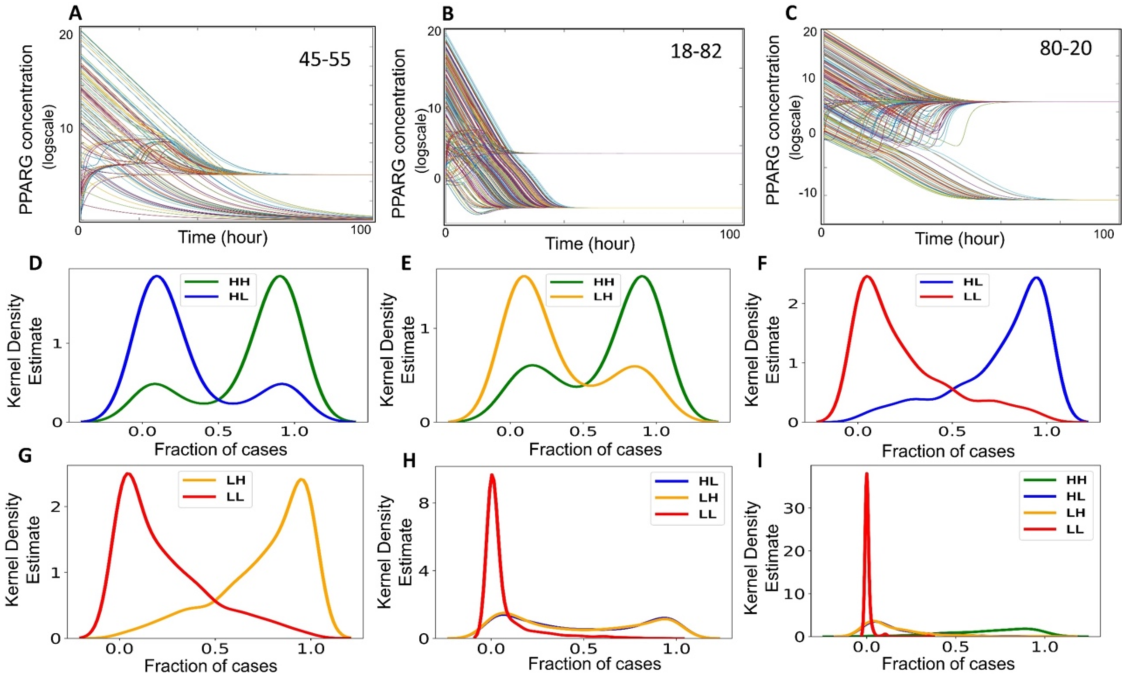

FIGURE S3: *Relative stability for multistable phases.* ***(A-C)*** *Schematic representation of relative stability: A system of coupled ordinary differential equations, when simulated for different parameter sets each enabling bistability (two states), can lead to different proportions of initial conditions converging to the two steady states.* ***(A)*** *45 initial conditions lead to state 1, 55 of them lead to state 2.* ***(B)****18 initial conditions lead to state 1, 82 of them lead to state 2.* ***(C)*** *80 initial conditions lead to state 1, 20 of them lead to state 2.* ***(D-I)*** *Kernel density estimates for relative stability of different states in the various multi-stable phases:* ***(D)*** *{HH, LH},* ***(E)*** *{HH, HL},* ***(F)*** *{HH, LL},* ***(G)*** *{LH, LL},* ***(H)*** *{HL, LH, LL},* ***(I)*** *{HL, LH, HH, LL}*

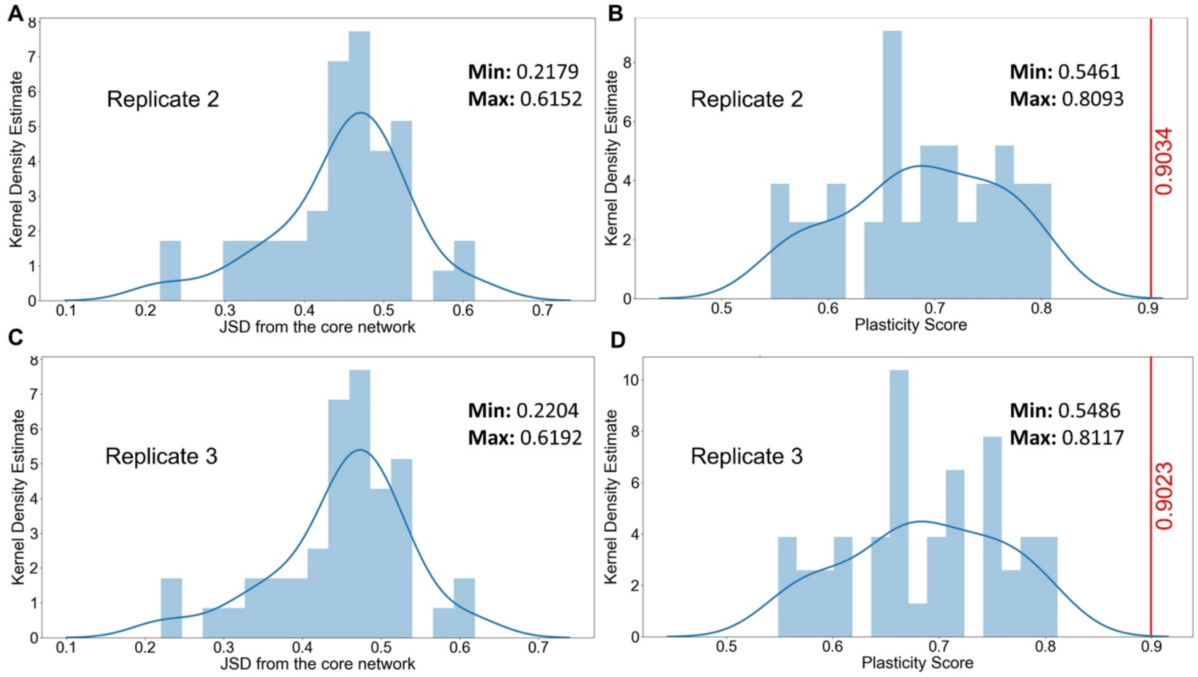

FIGURE S4: *Kernel density estimate plots. Jensen-Shannon Distance (JSD)* ***(A, C)*** *and Plasticity Scores* ***(B, D)*** *for two more RACIPE replicates apart from the one in the main figure 5B-C. In B, D; the red vertical line indicates the plasticity score (=0.90) of the "wild type" core regulatory circuit which is greater than all other "hypothetical" randomized networks.*

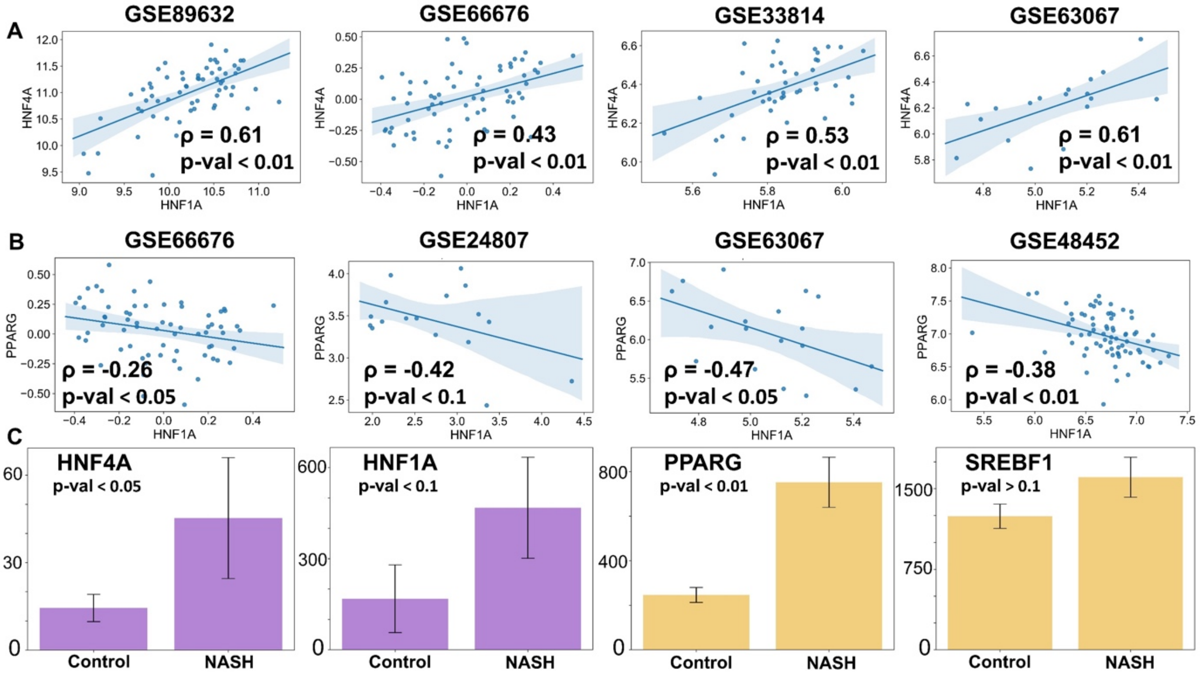

FIGURE S5: *Clinical data validation* ***(A)*** *Scatter plots between HNF1A and HNF4A in clinical samples.* ***(B)*** *Scatter plots between PPARG and SREBF1 in clinical samples.* ***(C)*** *Comparison of levels of HNF4A, HNF1A, PPARG and SREBF1 in liver sinusoid endothelial*

cells for control case vs. mouse model of NASH based on RNA-Seq data. Expression values have been listed as TPM (transcripts per million) for given RNA-seq data. Spearman correlation coefficient is given by ρ, and p-val denotes the corresponding p-value wherever mentioned.

A

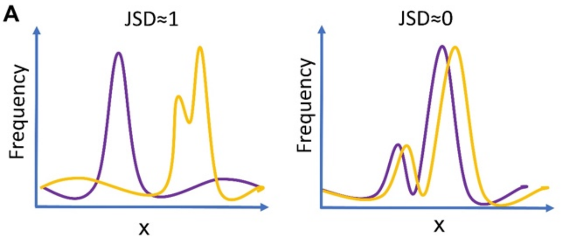

B

Condition*	JSD score
Euler 1000 initial conditions	0.0122 ± 0.0019
Euler 10000 initial conditions	0.0112 ± 0.0003
Runge-Kutta 100 initial conditions	0.0115 ± 0.0018

* with reference to Euler 100 initial conditions

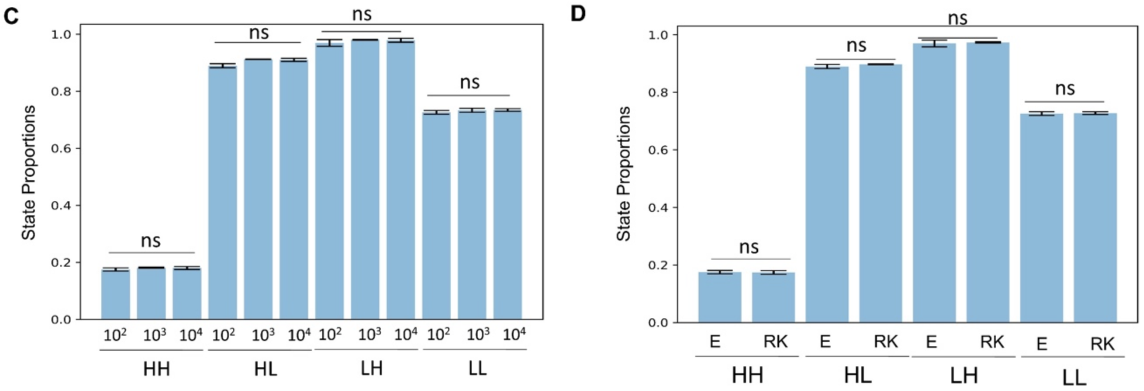

Figure S6: *Robustness of methods used.* ***(A)*** *Schematic describing JSD.* ***(B)*** *JSD scores between the frequency of states obtained by different analysis methods. Euler integration method with 100 initial conditions – the default setting for RACIPE – is considered as reference distribution and is used in the rest of this study.* ***(C)*** *States proportions of different states do not change significantly if the analysis is done with 100, 1000 or 10000 initial conditions.* ***(D)*** *State proportions do not change significantly whether E (Euler integration method) or RK (Runge-Kutta Integration method) is used. ns represents non-significant differences between the different simulation conditions, for students' t-test done between corresponding 3 replicates.*
